# Chronic Periodontitis is Associated With Cerebral Atherosclerosis –A Nationwide Study

**DOI:** 10.7759/cureus.11373

**Published:** 2020-11-07

**Authors:** Urvish K Patel, Preeti Malik, Nishanth Kodumuri, Priyadarshee Patel, Varun Pitti, Gaurav Tyagi, Bindi Chauhan, Abhishek Lunagariya, Ravish Kothari, Souvik Sen

**Affiliations:** 1 Neurology and Public Health, Icahn School of Medicine at Mount Sinai, New York, USA; 2 Public Health, Icahn School of Medicine at Mount Sinai, New York, USA; 3 Neurology, Massachusetts General Hospital, Andover, USA; 4 Neurology, Palmetto Health-University of South Carolina School of Medicine, Columbia, USA; 5 Neurology, Drexel University College of Medicine, Philadelphia, USA; 6 Dentistry, Rutgers School of Dental Medicine, Newark, USA; 7 Public Health, Long Island University, New York, USA; 8 Neurology, Creighton University School of Medicine, Omaha, USA

**Keywords:** nationwide inpatient sample (nis), chronic inflammation, cardiovascular disease, cerebral atherosclerosis, chronic periodontitis, cerebrovascular disease, atherosclerosis, acute ischemic stroke, hemorrhagic stroke, transient ischemic attack

## Abstract

Introduction

Chronic periodontitis and atherosclerosis share common risk factors and produce the same inflammatory markers. Many studies found a high prevalence of chronic periodontitis in patients with atherosclerosis but there is no strong evidence to support a specific association of chronic periodontitis with cerebral atherosclerosis. We aimed to study the concurrent prevalence and association of chronic periodontitis with cerebral atherosclerosis and cerebrovascular diseases among the US population.

Methods

We performed a cross-sectional analysis of a Nationwide Inpatient Sample with adult hospitalizations to identify the primary diagnosis of cerebrovascular diseases [acute ischemic stroke (AIS), hemorrhagic stroke (HS), and transient ischemic attack (TIA)] with concurrent cerebral atherosclerosis and chronic periodontitis. Multivariate survey logistic regression models were fitted to evaluate the linkage of chronic periodontitis with cerebral atherosclerosis and cerebrovascular diseases.

Results

Of total 56,499,788 hospitalizations, 0.01% had chronic periodontitis. Prevalence of chronic periodontitis was higher in 50-64 years (36.18% vs. 23.91%), males (59.19% vs. 41.06% in females), Afro-Americans (25.93% vs. 15.21%), and 0-25th percentile median-household-income-category (38.31% vs. 30.15%) compared to non-chronic periodontitis. There was significantly higher prevalence of cerebral atherosclerosis (0.71% vs. 0.41%; p<0.0001) with weak evidence of high prevalence of cerebrovascular diseases (AIS:2.21% vs. 1.97%; p=0.1563; HS:0.57% vs. 0.46%; p=0.1560) among chronic periodontitis compared to non-chronic periodontitis. In regression analysis, odds of having cerebral atherosclerosis were 2.48-folds higher in patients with chronic periodontitis compared to that without-chronic periodontitis, and cerebral atherosclerosis patients were associated with higher odds of TIA (aOR:2.40; p<0.0001), AIS (aOR:3.35; p<0.0001), and HS (aOR:1.51; p<0.0001) compared to without-cerebral atherosclerosis. No significant relationship between chronic periodontitis and cerebrovascular diseases was observed.

Conclusion

Although chronic periodontitis may not directly increase the risk of cerebrovascular diseases, it increases the burden of cerebrovascular diseases by evidently increasing the risk of cerebral atherosclerosis. Early identification of chronic periodontitis and atherosclerotic risk factors may help to mitigate the risk of cerebrovascular diseases.

## Introduction

Chronic periodontitis is a chronic inflammation caused by bacterial colonization that affects the periodontal tissue supporting the teeth. The prevalence of chronic periodontitis is high, affecting 90% of the global population and therefore contributes significantly to the global burden of chronic diseases [1,2]. Hence, chronic periodontitis is arising as a major public health issue. According to a recent report from the Centers for Disease Control and Prevention (CDC), 47.2% of adults >30 years of age have periodontitis which increases with age and 70.1% of people by >65 years of age have chronic periodontitis [3]. 

Chronic Periodontitis can be a risk factor for many systemic diseases like cardiovascular disease, carotid artery disease, cerebrovascular disease, etc. contributing significantly to mortality and morbidity worldwide due to common inflammatory pathogenesis [1,4,5]. Studies have shown that chronic periodontitis patients are prone to the dissemination of oral bacteria and endotoxins into the bloodstream, which can trigger a pro-atherogenic response in endothelial cells leading to systemic inflammation and fibrin deposition. Eventually, this increased systemic inflammatory response is associated with the onset and progression of atherosclerosis and cerebrovascular disease [6-9]. Chronic periodontitis shares many concurrent conditions and risk factors for vascular disorders like atherosclerosis, stroke, cardiovascular disease, hypertension, dyslipidemia, diabetes mellitus, and smoking [10].

The exact prevalence of periodontitis in a patient with cerebral atherosclerosis and cerebrovascular diseases is not known. Few meta-analyses including both retrospective and prospective studies have reported a higher risk of cardio- and cerebrovascular disease in patients with chronic periodontitis [5,11-14]. But subtypes of stroke such as acute ischemic stroke (AIS), hemorrhagic stroke (HS), and transient ischemic attack (TIA) have not been analyzed separately in those meta-analyses. Also, those meta-analyses were from small cohort observational studies, and the estimated association may not be true considering the influence of biases (selection and observer) and confounding factors. All these limitations with previous studies prompt the need for a large population-based study to determine the prevalence of chronic periodontitis in a large group of patients with cerebral atherosclerosis and cerebrovascular diseases.

In this cross-sectional retrospective study, we aimed to describe the prevalence of cerebral atherosclerosis and cerebrovascular diseases among a large cohort of patients with concurrent chronic periodontitis and to assess whether chronic periodontitis is associated with cerebral atherosclerosis and cerebrovascular diseases like AIS, HS, and TIA.

## Materials and methods

We have obtained Nationwide Inpatient Sample (NIS) data from the Healthcare Cost and Utilization Project (HCUP) between January 2013 and December 2014. The NIS is the all-payer inpatient care database in the US and contains discharge-level data provided by states that participate in the HCUP. This dataset acquires approximately 20% of the stratified sample of all US community hospitals, representing more than 95% of the national population. Each hospitalization is treated as an individual entry in the database and is coded with one principal diagnosis and up to 24 secondary diagnoses associated with that stay. Detailed information on NIS is available at http://www.hcup-us.ahrq.gov/db/nation/nis/nisdde.jsp. The NIS is a de-identified database, so informed consent or IRB approval was not needed for the study. The HCUP Data Use Agreement (HCUP-348L73IZS) for the data utilized in this study was obtained. [15]

Study population

We used the 9th revision of the International Classification of Diseases, clinical modification codes (ICD-9-CM) to identify adult patients admitted with a primary and secondary diagnosis of chronic periodontitis (ICD-9-CM code 523.4) [11]. Similarly, patients with concurrent diagnosis of cerebral atherosclerosis (atheroma of cerebral arteries; ICD-9-CM code 437.0), transient ischemic attack (ICD-9-CM code 435), acute ischemic stroke (ICD-9-CM codes 433.01, 433.11, 433.21, 433.31, 433.81, 433.91, 434.01, 434.11, 434.91), and hemorrhagic stroke (ICD-9-CM codes 430, 431) were identified. The ischemic stroke and transient ischemic attack codes have 35% sensitivity, 99% specificity, 96% PPV (positive predictive values), and 79% NPV (negative predictive values); hemorrhagic stroke codes have 60% sensitivity, >99% specificity, 77% PPV, and >99% NPV; and cerebrovascular diseases combined codes have 46.3% sensitivity, 99.1% specificity, 81.2% PPV, and 95.4% NPV [16]. We used ICD-9-CM codes to identify the comorbidities of diabetes mellitus, hypertension, obesity, hypercholesterolemia, current or past smoker, drug abuse/dependent, alcohol abuse/dependent, AIDS, renal failure (Acute/ Chronic/ ESRD). Table 1 lists all ICD-9-CM codes that were used for this study. Age <18 years and admissions with missing data for age, sex, and race were excluded.

**Table 1:** ICD-9-CM codes used in this analysis

Condition	ICD-9 CM Codes
HIV/AIDS	042, V08
Hypercholesterolemia	272.0, 272.1, 272.2
Obesity	278.00, 278.01, 278.02
Hypertension	401-405
Diabetes Mellites	249.00, 250
Alcohol abuse/dependent	V11.3, 303, 305.0
Current or past smoker	V15.82, 305.1
Drug abuse	304, 305.2-305.9
Renal failure (Acute/ Chronic/ ESRD)	584, 585, 585.6

Patient and hospital characteristics

Patient characteristics of interest were age, sex, race, insurance status, comorbidities, and concomitant diagnoses as defined above. The race was defined by white (referent), African American, Hispanic, Asian or Pacific Islander, and Native American. Insurance status was defined by Medicare (referent), Medicaid, Private Insurance, and Other/Self-pay/No charge. We defined the severity of co-morbid conditions using Deyo's modification of the Charlson co-morbidity index (CCI) (Table 2).

**Table 2:** Deyo’s modification of Charlson’s co-morbidity index (CCI)

Condition	ICD-9-CM Codes	Charlson Score
Myocardial infarction	410 – 410.9	1
Congestive heart failure	428 – 428.9	1
Peripheral vascular disease	433.9, 441 – 441.9, 785.4, V43.4	1
Cerebrovascular disease	430 – 438	1
Dementia	290 – 290.9	1
Chronic pulmonary disease	490 – 496, 500 – 505, 506.4	1
Rheumatologic disease	710.0, 710.1, 710.4, 714.0 – 714.2, 714.81, 725	1
Peptic ulcer disease	531 – 534.9	1
Mild liver disease	571.2, 571.5, 571.6, 571.4 –571.49	1
Diabetes	250 – 250.3, 250.7	1
Diabetes with chronic complications	250.4 – 250.6	2
Hemiplegia or paraplegia	344.1, 342 – 342.9	2
Renal disease	582 – 582.9, 583 – 583.7, 585, 586, 588 – 588.9	2
Any malignancy including leukemia and lymphoma	140-172.9, 174-195.8, 200-208.9	2
Moderate or severe liver disease	572.2 – 572.8	3
Metastatic solid tumor	196-199.1	6
AIDS	042	6

Outcomes

The primary aim of the study is to identify the prevalence of cerebral atherosclerosis and cerebrovascular diseases (TIA, AIS, and HS) among patients with concurrent chronic periodontitis. The secondary aim is to evaluate the association of cerebral atherosclerosis and cerebrovascular diseases with chronic periodontitis. 

Statistical analysis

All statistical analyses were performed using the weighted survey methods in SAS (version 9.4). Weighted values of patient-level observations were generated to produce a nationally representative estimate of the entire US population of hospitalized patients. P-values of <0.05 was considered significant. Univariate analysis of differences between categorical variables was tested using the chi-square test. The mixed-effects survey logistic regression models with weighted analysis were used for the dependent variables, in order to estimate odds ratio (OR) and 95% Confidence Interval for the evaluation of the relationship of chronic periodontitis with cerebral atherosclerosis and cerebrovascular diseases and the relationship of cerebral atherosclerosis with cerebrovascular diseases.

In the multivariable analysis, we included demographics (age, gender, race), patient-level hospitalization variables (admission day, primary payer, admission type, Median Household Income Category), hospital-level variables (hospital region, teaching versus nonteaching hospital, hospital bed size), comorbidities like diabetes mellitus, hypertension, obesity, hypercholesterolemia, current or past smoker, drug abuse/dependent, alcohol abuse/dependent, AIDS, renal failure (Acute/Chronic/End-stage renal disease or ESRD) and CCI.

For each model, c-index (a measure of goodness of fit for binary outcomes in a logistic regression model) was calculated. All statistical tests used were 2-sided, and p<0.05 was deemed statistically significant. No statistical power calculation was conducted prior to the study.

Data availability

The data that support the findings of this study are publicly available from the Agency for Healthcare Research and Quality's Healthcare Cost and Utilization Project (HCUP) Nationwide Inpatient Sample (NIS). A raw analysis of the data will be however made available from the authors upon request and with permission of HCUP-NIS.

## Results

Disease hospitalizations

There was a total of 56,499,768 hospitalizations in the United States from year January 2013 to December 2014 after excluding patients with age <18 years and admissions with missing data for age, gender, and race. Out of 56,499,768 hospitalizations, 7020 (0.01%) had chronic periodontitis.

Demographics, patient and hospital characteristics, and comorbidities

In the US hospitalizations, the prevalence of chronic periodontitis was higher in patients age group 50-64 years (36.18% vs. 23.91%, p<0.0001), male (59.19% vs. 41.06%, p<0.0001), African American (25.93% vs. 15.21%, p<0.0001), and 0-25th percentile median household income category (38.31% vs. 30.15%, p<0.0001) compared to non-chronic periodontitis. The frequency of chronic periodontitis was higher among patients with co-morbidities such as obesity (15.24% vs. 13.66%, p=0.0001), drug abuse (10.83% vs. 4.93%, p<0.0001), alcohol abuse (10.83% vs. 5.98%, p<0.0001), and current or past smoker (43.87% vs. 27.45%, p<0.0001) than those without chronic periodontitis. Hospitalizations with chronic periodontitis were also associated with a higher percentage of CCI (Deyo’s Charlson Co-morbidity) Index 2, 3, and ≥5. US Hospitalizations in large, urban teaching hospitals and in the Midwest and South regions have a higher prevalence of chronic periodontitis compared to non-chronic periodontitis (Table 3).

**Table 3:** Characteristics of chronic periodontitis in US hospitalizations (2013-2014) * Bedsize of hospital indicates the number of hospital beds which varies depending on hospital location (Rural/ Urban), teaching status (Teaching/ Non-teaching), and Region (Northeast/Midwest/ Southern/Western) The numbers in the table are column percentage (%) indicates a direct comparison between Chronic Periodontitis Vs. Non- Chronic Periodontitis amongst US hospitalizations between January 2013-December 2014.

	Chronic Periodontitis	Non-chronic Periodontitis	Total	p values
Weighted frequency (%)	7020 (0.01%)	56492768 (99.99%)	56499768 (100)	< 0.0001
Demographics of Patients				
Age Mean ±SD	53.2 ± 16.7	57.4 ± 20.5		
Age Group (Years) (%)				<0.0001
18 - 34	16.81	19.58	19.58	
35 - 49	22.08	14.81	14.81	
50 - 64	36.18	23.91	23.91	
65 - 79	18.23	24.95	24.95	
≥ 80	6.70	16.74	16.74	
Gender (%)				< 0.0001
Male	59.19	41.06	41.07	
Female	40.81	58.94	58.93	
Race (%)				< 0.0001
White	60.44	70.62	70.62	
African American	25.93	15.21	15.21	
Hispanic	11.21	10.96	10.96	
Asian or Pacific Islander	1.90	2.60	2.60	
Native American	0.51	0.61	0.61	
Characteristics of Patients				
Median Household Income Category for patient's Zip code (%)				<0.0001
0-25th percentile	38.31	30.15	30.15	
26-50th percentile	26.40	26.75	26.75	
51-75th percentile	20.88	23.21	23.21	
76-100th percentile	14.41	19.88	19.88	
Primary Payer (%)				< 0.0001
Medicare	35.62	46.74	46.73	
Medicaid	26.20	16.90	16.90	
Private Insurance	21.27	27.68	27.68	
Other/Self-pay/No charge	16.92	8.68	8.68	
Admission type (%)				<0.0001
Non- elective	85.20	75.46	75.47	
Elective	14.80	24.54	24.53	
Admission day (%)				0.0981
Weekday	78.92	79.71	79.71	
Weekend	21.08	20.29	20.29	
Characteristics of Hospitals				
Bedsize of hospital (%) *				<0.0001
Small	12.11	16.22	16.22	
Medium	21.01	28.05	28.05	
Large	66.88	55.73	55.73	
Hospital Location & Teaching Status (%)				0.0001
Rural	6.55	33.29	33.29	
Urban Non-teaching	19.09	33.29	33.29	
Urban Teaching	74.36	33.29	33.29	
Hospital Region (%)				< 0.0001
Northeast	18.09	20.10	20.10	
Midwest	23.15	20.49	20.49	
South	42.31	40.13	40.13	
West	16.45	19.27	19.27	
Comorbidities of Patients (%)				
Diabetes mellitus	25.36	25.42	25.42	0.8978
Hypertension	50.78	52.69	52.69	0.0014
Obesity	15.24	13.66	13.66	0.0001
Hypercholesterolemia	4.42	5.54	5.54	<0.0001
Drug Abuse	10.83	4.93	4.93	<0.0001
Alcohol Abuse	10.83	5.98	5.98	<0.0001
Current or Past smoker	43.87	27.45	27.45	<0.0001
Deyo's Charlson Comorbidity Index (CCI) (%)				< 0.0001
0				
1	55.70	63.45	63.45	
2	17.02	13.46	13.46	
3	10.11	8.37	8.37	
4	4.99	5.72	5.72	
≥ 5	12.81	9.00	9.00	

Outcomes of chronic periodontitis and cerebral atherosclerosis among US hospitalizations

In univariate analysis, there was higher prevalence of cerebral atherosclerosis among chronic periodontitis compared to non-chronic periodontitis (0.71% vs. 0.41%, p<0.0001). Though the prevalence of acute ischemic stroke (AIS) and hemorrhagic stroke (HS) were higher in patients with non- chronic periodontitis (AIS: 2.21% vs. 1.97%, p=0.1563; HS: 0.57% vs. 0.46%, p=0.1560), the evidences of significance were weak (Table 4). Patients with cerebral atherosclerosis have higher frequency of AIS (14.26% vs.1.92%, p<0.0001), TIA (3.55% vs. 0.68%, p<0.0001) and HS (1.48% vs. 0.45%, p<0.0001) compared to no cerebral atherosclerosis (Table 5).

**Table 4:** Univariate Analysis of the Outcomes amongst Chronic Periodontitis. The numbers in the table are column percentage (%) indicates a direct comparison of outcomes (concurrent diagnoses) between Chronic Periodontitis Vs. Non- Chronic Periodontitis among US hospitalizations between January 2013-December 2014.

	Chronic Periodontitis	Non-chronic Periodontitis	Total	p values
Weighted frequency (%)	7020 (0.01%)	56492768 (99.99%)	56499768 (100)	< 0.0001
Cerebral Atherosclerosis	0.71	0.41	0.41	< 0.0001
Acute Ischemic stroke (AIS)	2.21	1.97	1.97	0.1563
Transient Ischemic stroke (TIA)	0.57	0.69	0.69	0.2341
Hemorrhagic stroke (HS)	0.57	0.46	0.46	0.156

**Table 5:** Univariate Analysis of the Outcomes amongst Cerebral Atherosclerosis. The numbers in the table are column percentage (%) indicates a direct comparison of outcomes (concurrent diagnoses) between Cerebral Atherosclerosis Vs. no- Cerebral Atherosclerosis among US hospitalizations between January 2013-December 2014.

	Cerebral Atherosclerosis	No Cerebral Atherosclerosis	Total	p values
Weighted frequency (%)	229765 (0.41%)	56270023 (99.59%)	56499788 (100)	< 0.0001
Acute Ischemic stroke (AIS)	14.26	1.92	1.97	< 0.0001
Transient Ischemic stroke (TIA)	3.55	0.68	0.69	< 0.0001
Hemorrhagic stroke (HS)	1.48	0.45	0.46	< 0.0001

Regression model derivation

The overall adjusted odds for cerebral atherosclerosis in chronic periodontitis patients among US hospitalizations were 2.48 (95%CI:1.34-4.59, p<0.004), after adjusting for basic demographic with patient-level variables, comorbidities, CCI, concurrent conditions compared to non- chronic periodontitis patients (Table 6).

**Table 6:** Regression analysis showing association between chronic periodontitis with cerebral atherosclerosis UL: Upper Limit; LL: Lower Limit; ESRD: End Stage Renal Disease; AIDS: Acquired Immunodeficiency Syndrome; CCI: Deyo's Charlson Comorbidity Index The model is adjusted for basic demographic with patient-level variables, hospital-level variables such as hospital region, teaching status, and bed size, comorbidities, and CCI.

Variables	Odds Ratio (OR)	95% Confidence Interval (CI)		p values
							LL	UL	
No- Chronic Periodontitis	Reference			
Chronic Periodontitis	2.48	1.34	4.59	0.004
Age (Years)	1.05	1.05	1.06	<0.0001
Age groups (years)				
18 - 34	Reference			
35 - 49	3.01	2.38	3.81	<0.0001
50 - 64	5.08	4.02	6.42	<0.0001
65 - 79	6.24	4.86	8	<0.0001
≥ 80	5.77	4.43	7.53	<0.0001
Gender				
Female	Reference			
Male	1.08	1.05	1.1	<0.0001
Race				
White	Reference			
African American	1.82	1.77	1.87	<0.0001
Hispanic	1.06	1.02	1.11	0.002
Asian or Pacific Islander	1.16	1.09	1.24	<0.0001
Native American	0.9	0.77	1.06	0.216
Median Household Income Category for patient's Zip code				
0-25th percentile	Reference			
26-50th percentile	0.98	0.96	1.01	0.167
51-75th percentile	0.99	0.96	1.02	0.446
76-100th percentile	1	0.97	1.03	0.888
Primary Payer				
Medicare	Reference			
Medicaid	1.1	1.05	1.16	0
Private Insurance	0.71	0.69	0.74	<0.0001
Other/Self-pay/No charge	0.89	0.84	0.95	0
Admission type				
Non- elective	Reference			
Elective	0.53	0.51	0.54	<0.0001
Admission day				
Weekday	Reference			
Weekend	1.01	0.98	1.03	0.652
Bedsize of hospital				
Small	Reference			
Medium	1.02	0.99	1.05	0.198
Large	1.04	1.01	1.06	0.013
Hospital Location & Teaching Status				
Rural	Reference			
Urban Non-teaching	0.87	0.84	0.9	<0.0001
Urban Teaching	0.94	0.91	0.97	<0.0001
Hospital Region				
Northeast	Reference			
Midwest	1.22	1.18	1.25	<0.0001
South	1.21	1.17	1.24	<0.0001
West	1.05	1.02	1.09	0.005
Adjusted Comorbidities				
Diabetes mellitus	0.9	0.88	0.92	<0.0001
AIDS	0.11	0.09	0.15	<0.0001
Renal failure (Acute/ Chronic/ ESRD)	0.54	0.53	0.56	<0.0001
Current or Past smoker	0.68	0.66	0.69	<0.0001
Hypertension	1.49	1.45	1.53	<0.0001
Obesity	0.63	0.6	0.65	<0.0001
Hypercholesterolemia	1.04	1	1.08	0.03
Drug abuse/dependent	1.25	1.16	1.35	<0.0001
Alcohol abuse/dependent	1.29	1.22	1.36	<0.0001
CCI	1.38	1.38	1.38	<0.0001
c- index	0.855			

Table 7 exhibits a multivariate regression analysis showing the association of chronic periodontitis and cerebral atherosclerosis with cerebrovascular diseases (AIS, TIA, and HS). Cerebral atherosclerosis patients were associated with higher odds of TIA attack (aOR: 2.40; 95%CI: 2.28-2.53; p<0.0001), AIS (aOR: 3.35; 95%CI: 3.25-3.45; p<0.0001), and HS (aOR: 1.51; 95%CI: 1.39-1.64, p<0.0001) compared to patients without cerebral atherosclerosis, though there was no direct evidence of relationship between chronic periodontitis and cerebrovascular disorders (TIA- aOR: 0.99, p=0.980; AIS-aOR: 1.02; p=0.919; HS- aOR: 1.02, p=0.956 for Model 1, 2, and 3, respectively).

**Table 7:** Regression analysis showing relationship of chronic periodontitis and cerebral atherosclerosis with cerebrovascular diseases TIA: Transient Ischemic Attack; AIS: Acute Ischemic Stroke; HS: Hemorrhagic Stroke OR: Odds Ratio; CI: 95% Confidence Interval; UL: Upper Limit; LL: Lower Limit; ESRD: End-Stage Renal Disease; AIDS: Acquired Immunodeficiency Syndrome; CCI: Deyo's Charlson Comorbidity Index The model is adjusted for basic demographic with patient-level variables, hospital-level variables such as hospital region, teaching status, and bed size, comorbidities, and CCI.

	Model 1: TIA				Model 2: AIS				Model 3: HS			
Variables	OR	95% CI		p values	OR	95% CI		p values	OR	95% CI		p values
		LL	UL			LL	UL			LL	UL	
No- Chronic Periodontitis	Reference											
Chronic Periodontitis	0.99	0.49	1.99	0.980	1.02	0.69	1.50	0.919	1.02	0.51	2.06	0.956
Cerebral Atherosclerosis (CA)	2.40	2.28	2.53	<0.0001	3.35	3.25	3.45	<0.0001	1.51	1.39	1.64	<0.0001
Age (Years)	1.02	1.02	1.02	<0.0001	1.03	1.03	1.03	<0.0001	1.01	1.01	1.01	<0.0001
Age groups (years)												
18 - 34	Reference											
35 - 49	3.95	3.67	4.26	<0.0001	2.49	2.39	2.60	<0.0001	2.28	2.14	2.42	<0.0001
50 - 64	4.20	3.85	4.59	<0.0001	2.75	2.61	2.89	<0.0001	2.42	2.23	2.64	<0.0001
65 - 79	3.98	3.57	4.43	<0.0001	2.63	2.47	2.80	<0.0001	2.62	2.34	2.92	<0.0001
≥ 80	3.85	3.39	4.37	<0.0001	2.46	2.28	2.65	<0.0001	2.56	2.23	2.95	<0.0001
Gender												
Female	Reference											
Male	0.92	0.91	0.93	<0.0001	1.13	1.12	1.14	<0.0001	1.14	1.12	1.16	<0.0001
Race												
White	Reference											
African American	1.10	1.08	1.13	<0.0001	1.21	1.19	1.22	<0.0001	1.07	1.04	1.10	<0.0001
Hispanic	1.06	1.03	1.08	<0.0001	0.91	0.90	0.93	<0.0001	1.11	1.07	1.15	<0.0001
Asian or Pacific Islander	0.77	0.72	0.81	<0.0001	1.16	1.13	1.20	<0.0001	1.74	1.67	1.82	<0.0001
Native American	0.97	0.86	1.08	0.529	0.91	0.85	0.97	0.004	1.01	0.88	1.15	0.9012
Median Household Income Category for patient's Zip code												
0-25th percentile	Reference											
26-50th percentile	1.00	0.98	1.02	0.719	1.02	1.01	1.04	0.0002	1.09	1.06	1.11	<0.0001
51-75th percentile	1.01	0.99	1.03	0.467	1.00	0.99	1.01	0.884	1.08	1.05	1.11	<0.0001
76-100th percentile	0.96	0.94	0.98	0.001	0.97	0.96	0.99	<0.0001	1.11	1.08	1.14	<0.0001
Primary Payer												
Medicare	Reference											
Medicaid	0.84	0.82	0.87	<0.0001	1.09	1.07	1.11	<0.0001	1.16	1.12	1.20	<0.0001
Private Insurance	1.07	1.05	1.10	<0.0001	1.27	1.25	1.28	<0.0001	1.40	1.36	1.44	<0.0001
Other/Self-pay/No charge	1.11	1.07	1.15	<0.0001	1.60	1.57	1.63	<0.0001	1.67	1.61	1.74	<0.0001
Admission type												
Non- elective	Reference											
Elective	0.19	0.19	0.20	<0.0001	0.26	0.26	0.27	<0.0001	0.28	0.27	0.30	<0.0001
Admission day												
Weekday	Reference											
Weekend	1.11	1.09	1.13	<0.0001	1.13	1.12	1.14	<0.0001	1.18	1.16	1.21	<0.0001
Bedsize of hospital												
Small	Reference											
Medium	1.06	1.03	1.08	<0.0001	1.15	1.13	1.16	<0.0001	1.70	1.61	1.79	<0.0001
Large	1.01	0.99	1.03	0.253	1.22	1.21	1.24	<0.0001	3.77	3.60	3.96	<0.0001
Hospital Location & Teaching Status												
Rural	Reference											
Urban Non-teaching	1.09	1.06	1.12	<0.0001	1.05	1.03	1.06	<0.0001	1.70	1.61	1.79	<0.0001
Urban Teaching	0.96	0.93	0.98	0.001	1.21	1.19	1.23	<0.0001	3.77	3.60	3.96	<0.0001
Hospital Region												
Northeast	Reference											
Midwest	0.98	0.96	1.00	0.083	1.08	1.06	1.10	<0.0001	1.08	1.04	1.11	<0.0001
South	1.11	1.08	1.13	<0.0001	1.22	1.21	1.24	<0.0001	1.20	1.17	1.23	<0.0001
West	0.97	0.94	0.99	0.008	1.20	1.18	1.21	<0.0001	1.27	1.23	1.31	<0.0001
Adjusted Comorbidities												
Diabetes mellitus	0.98	0.96	1.00	<0.0001	0.86	0.85	0.87	<0.0001	0.55	0.53	0.56	<0.0001
AIDS	1.11	1.08	1.13	<0.0001	0.19	0.18	0.21	<0.0001	0.14	0.12	0.17	<0.0001
Renal failure (Acute/ Chronic/ ESRD)	0.97	0.94	0.99	<0.0001	0.40	0.40	0.41	<0.0001	0.39	0.38	0.40	<0.0001
Current or Past smoker	0.98	0.96	1.00	<0.0001	0.95	0.94	0.96	<0.0001	0.73	0.71	0.74	<0.0001
Hypertension	1.11	1.08	1.13	<0.0001	2.12	2.09	2.14	<0.0001	2.20	2.14	2.25	<0.0001
Obesity	0.97	0.94	0.99	<0.0001	0.83	0.82	0.84	<0.0001	0.71	0.69	0.73	<0.0001
Hypercholesterolemia	0.98	0.96	1.00	<0.0001	1.21	1.19	1.23	<0.0001	0.90	0.86	0.93	0.030
Drug abuse/dependent	1.11	1.08	1.13	<0.0001	1.06	1.03	1.09	<0.0001	1.24	1.19	1.30	<0.0001
Alcohol abuse/dependent	0.97	0.94	0.99	<0.0001	0.85	0.83	0.87	<0.0001	0.98	0.94	1.02	0.2436
CCI	1.17	1.17	1.18	<0.0001	1.31	1.31	1.31	<0.0001	1.32	1.32	1.32	<0.0001
c- index	0.772				0.803				0.798			

Accuracy of the model

c-statistic was used to validate the accuracy of the regressions. It was 0.772, 0.803, 0.798 for adjusted model 1, 2 and 3 respectively. All models have c-index >0.7, which indicates a good model fit.

## Discussion

With increasing prevalence, chronic periodontitis is arising as a major public health concern contributing to the global burden of chronic diseases. According to the CDC, nearly half of the Americans aged 30 or older have periodontitis [3]. Chronic inflammation due to periodontal disease has a causal relation to atherosclerosis mediated by changes in inflammation and the immune response, with subsequent causative relationships to stroke and heart disease [17,18]. Prior observational studies have shown that poor periodontal health status is associated with increased stroke risk. In our large population-based cross-sectional study, we aimed to investigate the link between chronic periodontitis and cerebrovascular disease. Further assessment of risk profiles for periodontal disease in adults in the United States shows male sex, current cigarette smoking, and diabetes mellitus as important risk factors for periodontal disease [19]. 

Chronic periodontitis represents a systemic burden of bacteria, endotoxin, and other bacterial products, which can induce an abundant production of proinflammatory cytokines leading to inflammatory cell proliferation into large arteries and stimulate the hepatic synthesis of clotting factors, thus contributes to atherogenesis and thromboembolic events [1,20,21]. Chronic periodontal disease patients have increased levels of inflammatory cytokines like TNF - alpha, IL- 1, and IL- 6 which leads to fibrin deposition and atherogenic trigger response [1,22]. These hypotheses of pathogenesis help to establish the association of the periodontal disease with cardiovascular disease, cerebrovascular disease, and atherosclerosis. This may explain our study results of the increased incidence of atherosclerosis in chronic periodontitis.

In this large population-based cross-sectional study we found a direct association between chronic periodontitis with atherosclerosis and in-direct relation to a higher incidence of cerebrovascular disease. US hospitalizations with chronic periodontitis had a higher prevalence in the age group 50-64 years, male, African American, and 0-25th percentile median household income category patients compared to non-chronic periodontitis. Various studies have data on the higher prevalence of chronic periodontitis in African Americans due to poor dental hygiene [23]. In our study, chronic periodontitis was also associated with other comorbidities such as diabetes, hypertension, hyperlipidemia, obesity, drug abuse, alcohol abuse, and tobacco smoking. Interestingly, we found a 2.48 times more risk of cerebral atherosclerosis among chronic periodontitis as compared to no chronic periodontitis. Higher prevalence of AIS, TIA, and HS were noted in chronic periodontitis, but the evidence was not significant (p=0.1560). Higher prevalence of AIS, TIA, and HS are seen in patients with cerebral atherosclerosis as compared to patients without atherosclerosis, with odds of 3.35, 2.40, 1.51 in AIS, TIA, and HS respectively. In our study, we could not establish a direct relationship between cerebrovascular diseases and chronic periodontitis. Therefore, chronic periodontitis does not directly increase the risk of cerebrovascular diseases but increases the risk of cerebral atherosclerosis which is associated with increased risk of cerebrovascular diseases.

Several observational meta-analysis studies have shown that chronic periodontitis and poor periodontal health status is associated with an increased risk for stroke and cardiovascular diseases [24,25]. A large population-based, nationwide study in Taiwan identified the periodontal disease as an important risk factor for ischemic stroke incidence and showed that periodontal treatment lowered the risk of stroke, significantly among young adults [26]. Grau et al. carried out a case-control study in the German population, in which increasing severity of periodontitis was associated with an increased risk of ischemic stroke after adjustment for age, gender, number of teeth, and other covariables [27]. A more recent meta-analysis of two cohort studies found that periodontal disease increased the risk of ischemic strokes by 1.6-fold [5].

It is possible that an increased risk of thrombotic stroke may be secondary to atherothrombosis in the cervicocerebral vasculature and cardioembolic stroke association may be because of coronary artery disease or atrial fibrillation related to periodontal disease-induced inflammation. A recent study by Sen et al. cohort of the ARIC database was studied over a period of 15 years which demonstrated an increased hazard of cardioembolic and thrombotic stroke in chronic periodontitis. The study also shows regular dental care use continued to be associated with lower rates of ischemic stroke [28].

Our study analysis showed no direct linkage between cerebrovascular diseases and chronic periodontitis, but chronic periodontitis was significantly associated with an increased prevalence of cerebrovascular diseases. This contradictory finding may have arisen as periodontitis is usually managed on an outpatient basis, so inpatient data of chronic periodontitis prevalence might be underrepresented. There is a need to be more vigilant in diagnosing chronic periodontitis on both inpatient and outpatient basis. Early detection of chronic periodontitis, treatment, and stressing the need for dental hygiene can be emphasized which ultimately will lead to a reduction in the risk of cerebrovascular events.

We strongly recommend that chronic periodontitis should be identified as an important risk factor for cerebral atherosclerosis and a possible risk for cerebrovascular diseases (AIS, TIA, and HS). Poor oral hygiene which contributes to periodontal disease is a modifiable risk factor. Evidence from randomized controlled trials has established that intensive periodontal treatment improves systemic inflammation, high blood pressure, lipid profile, and endothelial dysfunction [29,30], further reducing the risk of atherosclerotic disease. Frequent tooth brushing and regular dental care will potentially reduce the risk of cerebrovascular diseases.

Strength and Limitations

To the best of our knowledge, this would be the first US-based large population cross-sectional study to report the relationship between chronic periodontitis and cerebral atherosclerosis. NIS Data represent the largest inpatient database. This study does have some limitations. We were not able to establish a direct causal relationship between chronic periodontitis and cerebrovascular disease with good statistical power. But this study successfully puts forward an indirect relationship between chronic periodontitis and cerebrovascular diseases. Diagnosis of chronic periodontitis in the NIS database is the physician-documented diagnosis, hence susceptible to coding errors, and with chronic periodontitis being usually managed in an outpatient setting, might have been underdiagnosed as a hospital problem. We were not able to quantify the severity of chronic periodontitis.

## Conclusions

Our study found a significant association of cerebral atherosclerosis with chronic periodontitis. Furthermore, we could not establish the directly associated risk of chronic periodontitis with cerebrovascular disease, but it increases the burden of cerebrovascular diseases through evidently increasing the risk of cerebral atherosclerosis. Early identification of chronic periodontitis and atherosclerotic risk factors helps to mitigate the risk of cerebrovascular diseases.
